# On the Misinterpretation of Fluorescence‐Based Quantification in Theranostic Corneal Cross‐Linking

**DOI:** 10.1002/jbio.70288

**Published:** 2026-05-28

**Authors:** Ciro Caruso, Gaetano Barbaro

**Affiliations:** ^1^ Eye Department Pellegrini Hospital ASL NA 1

**Keywords:** corneal cross linking, fluorescenceimaging, light‐tissue interaction, photochemical kinetics, riboflavin

To the Editor,

1

We read with great interest the study by Lombardo et al. proposing a theranostic UV‐A device for real‐time assessment of intracorneal riboflavin concentration during corneal cross‐linking (CXL) [1]. While the integration of diagnostic feedback into treatment delivery represents an important technological advance, several fundamental assumptions underlying this approach warrant critical reconsideration.

The central premise of the study is that fluorescence intensity can be used as a quantitative surrogate for intracorneal riboflavin concentration. However, fluorescence emission in biological tissues is not solely determined by fluorophore concentration but is strongly influenced by multiple confounding factors, including light scattering, absorption heterogeneity, and dynamic changes in tissue optical properties [2, 3]. In the corneal stroma, these factors evolve continuously during UV‐A irradiation due to hydration changes and structural modification, directly affecting both excitation and emission pathways.

Furthermore, riboflavin is an active photosensitizer undergoing continuous photodegradation and participating in oxygen‐dependent photochemical reactions. These processes introduce non‐linear kinetics and concentration‐dependent quenching effects, preventing a stable and direct relationship between fluorescence intensity and actual stromal concentration [4, 5]. Under such dynamic conditions, fluorescence should be considered a composite optical signal rather than a quantitative biomarker.

The limitations of this assumption are reflected in the reported agreement between fluorescence‐based measurements and spectrophotometric analysis, which deteriorates during UV‐A exposure—the very phase in which real‐time monitoring is intended to provide clinically meaningful information [1]. The factors invoked to explain this discrepancy, such as stromal thinning and increased scattering, are intrinsic to the CXL process and therefore represent fundamental constraints rather than correctable sources of error.

More broadly, the study highlights a critical methodological issue: the attempt to control a complex photochemical process using indirect optical signals that are not uniquely related to the underlying state variables. Corneal cross‐linking is governed by the interaction between UV‐A irradiance, riboflavin photochemistry, and oxygen availability, with kinetics that are highly sensitive to local environmental conditions [4, 6]. None of these variables are directly measured by fluorescence.

In contrast, approaches based on first‐principles modeling of riboflavin photodepletion allow the system to be described through deterministic kinetic equations. The temporal evolution of riboflavin concentration (*C*(*t*)) under UV‐A irradiation can be expressed as follows:

$$\mathrm{dC}\left(t\right)/\mathrm{dt}=-\sigma \;I\;C\left(t\right)$$

with solution:

$$C\left(t\right)=C₀e^{\left(-\sigma \;I\;t\right)}$$

where (*I*) is the irradiance and (\sigma) the effective photochemical cross‐section. This formulation demonstrates that the process is governed by fluence rather than instantaneous measurements and can be predicted from initial conditions without reliance on indirect optical surrogates. In this context, the use of fluorescence‐based feedback risks introducing an apparent level of control that is not supported by the underlying physics. The ability to measure a signal does not imply that the measured quantity is physically meaningful or sufficient to guide treatment. In conclusion, while theranostic CXL represents an innovative concept, the quantitative interpretation of fluorescence as a measure of intracorneal riboflavin concentration remains insufficiently substantiated. A more rigorous framework based on validated photochemical models and measurable physical parameters is required to achieve true treatment standardization.

## Funding

The authors have nothing to report.

## Conflicts of Interest

The authors declare no conflicts of interest.

## Data Availability

Data sharing is not applicable to this article as no datasets were generated or analyzed during the current study.

## References

[1] G. Lombardo , V. Villari , N. Micali , et al., “Non‐Invasive Optical Method for Real‐Time Assessment of Riboflavin Concentration in Corneal Stroma During UV‐A Irradiation,” Journal of Biophotonics 11 (2018): e201800028. 10.1002/jbio.201800028 29451741

[2] J. R. Lakowicz , Principles of Fluorescence Spectroscopy (Springer, 2006).

[3] S. L. Jacques , “Optical Properties of Biological Tissues: A Review,” Physics in Medicine and Biology 58 (2013): R37–R61. 10.1088/0031-9155/58/11/R37 23666068

[4] P. Kamaev , M. D. Friedman , E. Sherr , and D. Muller , “Photochemical Kinetics of Corneal Cross‐Linking With Riboflavin,” Investigative Ophthalmology & Visual Science 53 (2012): 2360–2367. 10.1167/iovs.11-9385 22427580

[5] J. Wernli , S. Schumacher , E. Spoerl , and M. Mrochen , “The Efficacy of Corneal Cross‐Linking Shows a Sudden Decrease With Very High Intensity UV Light and Short Treatment Time,” Investigative Ophthalmology & Visual Science 54 (2013): 1176–1180. 10.1167/iovs.12-11409 23299484

[6] A. S. McCall , S. Kraft , H. F. Edelhauser , et al., “Mechanisms of Corneal Tissue Cross‐Linking,” Investigative Ophthalmology & Visual Science 51 (2010): 129–138. 10.1167/iovs.09-3738 PMC2869064 19643975

